# Inspiratory Muscle Strength and Cardiorespiratory Fitness Association With Health-Related Quality of Life in Healthy Older Adults

**DOI:** 10.3389/fspor.2021.624947

**Published:** 2021-03-18

**Authors:** Ainoa Roldán, Pablo Monteagudo, Ana Cordellat, Gema Sanchis-Soler, Cristina Blasco-Lafarga

**Affiliations:** ^1^Sport Performance and Physical Fitness Research Group (UIRFIDE), Department of Physical Education and Sports, University of Valencia, Valencia, Spain; ^2^Department of Education and Specific Didactics, Jaume I University, Castellón de la Plana, Spain; ^3^Department of Education and Specific Didactics, University of Alicante, Alicante, Spain

**Keywords:** aging, multicomponent exercise program, physical function, respiratory system, well-being aging

## Abstract

The main purpose of this study was to explore similarities and differences in the association between two capabilities affecting the cardiorespiratory system (overall and multifactorial cardiorespiratory fitness and inspiratory muscle strength) and the health-related quality of life (HRQoL), in a group of active healthy seniors. Sixty-five individuals (age, 73.01 ± 5.27 years; 53 women) who participated regularly in a multicomponent training program completed the EuroQol 5D-5L questionnaire, the 6-min walking test (6MWT), and the maximum inspiratory pressure test (MIP). Non-parametric correlations (Spearman's rho) were conducted to analyze the association between HRQoL indices (EQindex and EQvas), MIP, and 6MWT, considering both, the whole sample and men and women separately. Furthermore, partial correlation was made by controlling age and sex. We found a moderate association between HRQoL and cardiorespiratory fitness (EQvas: *r* = 0.324, *p* = 0.009; EQindex: *r* = 0.312, *p* = 0.011). Considering sex, relationship EQvas-6MWT decrease to small (*r* = 0.275; *p* = 0.028) whereas EQindex-6MWT remained moderated (*r* = 0.425; *p* = 0.000). When we considered women and men separately, the association between HRQoL and 6MWT appeared only in women, while the observed strong trend (*p* = 0.051) toward a large and positive association between EQindex and MIP, mediated by the covariate age, appeared only in men. Conversely to the cardiorespiratory fitness, MIP is not a limiting factor of HRQoL in healthy active elderly. Moreover, MIP and HRQoL should be included in the assessment of exercise interventions because they provide different information about the cardiorespiratory system deterioration. Similarly, EQvas and EQindex confirm to be complementary in the assessment of HRQoL. Furthermore, like aging process is different for men and women, the association between MIP and cardiorespiratory fitness with HRQoL may behave differently, so keeping on research these associations could help to improve training programs for this population.

## Introduction

Cardiorespiratory fitness (CRF) is a predictor of mortality and comorbidity, regardless of race and sex (DeFina et al., 2015; Strasser and Burtscher, 2018). It is positively associated to better functional capacity (Mandsager et al., 2018; Tomás et al., 2018) and thus to higher health-related quality of life (HRQoL) (Ciprandi et al., 2018; Ihász et al., 2020). Moreover, it has shown to be inversely related to cardiovascular disease as well as chronic pathologies that can affect healthy senior adults (DeFina et al., 2015; Bouaziz et al., 2018, 2019).

This multifactorial predictor is severely affected by the aging process, which involves alterations and adaptations in all body systems (Vilaça et al., 2019), with special attention to the decline in muscle mass (Cruz-Jentoft et al., 2019) and muscle strength (Blasco-Lafarga et al., 2020). These two factors, muscle mass and muscle strength, follow similar, but not equal impairment processes (Blasco-Lafarga et al., 2021). Shaw et al. (2017) reviewed the epidemiology of sarcopenia attending to the similarities and differences between the patterns of variation with age, gender, geography, time, and individual risk factors. They conclude that the rate of decline in muscle mass is much less rapid than the rate in muscle strength. These losses imply a reduction in functional capacity and activities of daily living (Manini and Clark, 2012; Tsekoura et al., 2017; Wang et al., 2020), both considered determinant factors in the HRQoL in older people. Higher functional capacity levels are related to higher HRQoL (Ran et al., 2017; de Oliveira et al., 2019), and this is of paramount importance, especially in women, who display worse results on HRQoL and functional capacity than men of the same age due to a higher prevalence of disability and chronic conditions (Orfila et al., 2006). Furthermore, aging process is different for men and women. Overall, men, who have higher values in both bioenergetics and neuromuscular capacities, refer greater losses with age (Botoseneanu et al., 2016; Riddle et al., 2018), as well as less life expectancy (Ventura-Clapier et al., 2017; Crimmins et al., 2019). Particularly in the CRF, Eriksen et al. (2016) analyzed 16,025 adults ranging from 18 to 91 years and found that men lost 0.26 ml/min/kg/year while women lost 0.23 ml/min/kg/year. Already in 1994, Enright pointed out that inspiratory muscle strength was 30% higher in men than in women, and men losses were also larger (Enright et al., 1994).

On the one hand, the association between lung function and health-related quality of life has previously been studied in older adults with pathology, but the effects of lung function on HRQoL among general population is not clear (Wen et al., 2019). The progressive reduction in thoracic wall compliance, lung elastic recoil, and loss of strength in respiratory muscles (Janssens, 2005), related to age, lead to an increment in dyspnea during daily living activities, limiting physical activity or exercise performance (Mills et al., 2015), and also influencing quality of life (Wen et al., 2019). These authors suggested that lower values in FVC and FEV1 were associated with decreased scores in total HRQoL and physical domain in participants without comorbidities. According to recent studies, respiratory muscle weakness alone becomes a major limiting factor for physical fitness improvement, since it triggers a reduction in general muscle strength, dyspnea, and changes in lung function (Vilaça et al., 2019). This fact indicates that changes in inspiratory muscle strength (a variable little used in the field of healthy older adults), could detect respiratory diseases earlier than spirometric values (Schoser et al., 2017), being important to know the optimal maximum inspiratory force values for each age group, even in the absence of pathology. Recently, our research group (Roldán et al., 2019) has confirmed a moderate and positive association between the maximum inspiratory pressure (MIP) and the CRF in a recent study with healthy older adults, and this association has shown to be modified by the covariates age and sex.

On the other hand, in the last decades, the prevention policies and health promotion in the field of seniors have become a main concern for administrations and healthcare professionals (Monteagudo et al., 2020); a link to avoid disease and disability, maintaining high physical and cognitive function, and sustaining engagement in social and productive activities (Ciprandi et al., 2018). Active aging and quality of life are thus main targets in these health policies, and HRQoL arises as a key factor in the analysis of their impact, even in pathologic situation or to assess any treatment benefit (Huang et al., 2011). Its multidimensional approach and the inclusion of physical, mental, and social aspects (Ciprandi et al., 2018), explain its relevance.

In this context, the present study aims to explore similarities and differences in the association between the HRQoL and two capabilities affecting the cardiorespiratory system (the overall CRF and the more local MIP), in a group of active healthy seniors. The influence of age and sex will also be considered. According to our previous studies, we hypothesize that people who present higher values in CRF will have greater HRQoL. In addition, we expect that higher inspiratory muscle strength will also be related to higher HRQoL, although the level of this association may be different, because the inspiratory strength requires a small neuromuscular and physiological response compared with the multifactorial assessment of the CRF.

## Materials and Methods

### Participants

Sixty-five active Spanish healthy elderly (53 women) volunteered to participate in this cross-sectional study approved by the local research ethics committee (H1506353751695). Previously, all of them were fully informed about the experimental procedure and signed the written consent. Inclusion criteria were as follows: (1) to be over 60 years old and (2) to participate regularly in the multicomponent training program EFAM-UV^©^ (thus physically and cognitively able to follow the training program, with no medical contraindications). Exclusion criteria were as follows: (1) to be smoker; (2) to have heart or respiratory disease; (3) to have hypertension; and (4) to suffer from retinopathies, adverse pharmacological treatments, ribcage disease, fatigue, pain, or illness.

### Measurements

#### Health-Related Quality of Life

HRQoL was assessed using the Spanish version of the EuroQuol 5D-5L questionnaire (EQ-5D-5L) (Herdman et al., 2011). This questionnaire comprises five dimensions (mobility, self-care, daily activities, pain/discomfort, and anxiety/depression). Each dimension is assessed on a single question with five response levels (EQindex; utility index) using five possible levels of problem (1 = no problems; 2 = slight problems; 3 = moderate problems; 4 = severe problems; 5 = unable/extreme problems). The five-dimension scores can be combined, and 3,125 possible health states are thus obtained. These health scores can be converted into a utility index ranging from −1 to +1, by applying the appropriate formula. An index score of 1 means perfect health state, and it coincides with the value 11,111. On the other hand, in Spanish population, the worst health corresponds to −0.654, and with the 5-digit number 55,555 (Herdman et al., 2011; Kim et al., 2013; Mateo et al., 2015).

Furthermore, EuroQol includes a standard visual analog scale (VAS; EQvas) in which respondents rate their overall health using a scale from 0 to 100. Participants answered the questionnaire themselves. Assistance was limited to rereading questions slowly when required.

#### Inspiratory Muscle Strength

Inspiratory muscle strength was measured by MIP test using an electronic device Powerbreathe® K5 (Powerbreathe K5, HaB International Ltd. UK). Following the protocol of Neder et al. (1999), participants were seated in a chair with their feet on the floor and their backs straight, and repeated three forced inspiration with a nose clamp. If the difference between these three inspirations was > 10%, up to five measurements could be taken. The best value was retained for further analysis.

#### Cardiorespiratory Fitness

CRF was measured by means of the 6MWT. Recently, Sperandio et al. (2019) compared the main physiological variables in 6MWT with the same variables in cardiopulmonary exercise testing (CPET) in healthy middle-age and older adults. They conclude that 6MWT is valid for assessing CRF in this population since there is a strong correlation between peaks VO_2_ in the 6MWT and in the CPET.

Attending to the protocol from Rikli and Jones (1999), the senior participants walked as fast as possible without running around a rectangle of 20 × 5 m, with signaling cones each 5 m. They were encouraged during the test and were informed every minute for the time. At the end, technician noted the total distance covered.

### Experimental Procedure

The assessments were carried out during three testing days, separated from 2 to 5 days. On their 1st day, the participants came to the lab and fulfill the HRQoL questionnaire. Then they were evaluated of body composition, blood pressure, arterial oxygen saturation, and maximum inspiratory pressure. On the 2nd day, they performed some strength tests like the hand grip test and the seat to stand test, not included in this study. On their 3rd day, they performed the 6MWT to assess the CRF.

### Statistical Analysis

Data were analyzed with Statistical Package for the Social Sciences, SPSS v26 for Mac (IBM Inc. Chicago, USA). After testing for normality (Kolmogorov-Smirnov for the total sample and women; Saphiro-Wilk for men), the Mann–Whitney *U*-test for non-parametric mean comparisons, and the nonparametric correlation analysis (Spearman's rho) were conducted to analyze the association between HRQoL indices (EQindex and EQvas) and the cardiorespiratory outcomes (maximum inspiratory pressure and 6MWT). The analyses were first performed considering the whole sample, followed by partial correlation controlling by age (*r*^a^), sex (*r*^s^), and sex+age (*r*^s+a^). Later on, the correlation analyses were repeated considering women (*r*^w^) and men (*r*^m^) separately, now only controlling for the age. Significance was considered *p* < 0.05. To assess the degree of these associations, we considered: *r* < 0.1, trivial; 0.1–0.3, small; 0.3–0.5, moderate; 0.5–0.7, large; 0.7–0.9, very large; >0.9, almost perfect; and 1 perfect (Hopkins, 2002). To add information about associations, scatter plot of *z*-scores and *R*^2^ were included as a measure of the effect size following Sullivan and Feinn (2012).

## Results

### Sample Characteristics and Differences by Sex

Sample characteristics are described in Table 1. There were sex differences in all the variables except in age, BMI, oxygen arterial saturation, and quality of life (EQvas and EQindex). Men and women show CRF scores above the mean, for their age group, according to Rikli and Jones (2013), specifically, 14.10% for men and 9.87% for women. However, despite this values, the MIP outcomes were discrete, similar to previous studies in the older adults following the EFAM-UV^©^ program (Roldán et al., 2019; Blasco-Lafarga et al., 2021) or similar interventions with Pilates (Alvarenga et al., 2018).

**Table 1:** Sample characteristics.

	**Total (*n* = 65)**	**Women (*n* = 53; 81.54%)**	**Men (*n* = 12; 18.46%)**	***p*-value**
	**Mean ± SD**	**Mean ± SD**	**Mean ± SD**	
**Anthropometric variables**				
Age (years)	73.01 ± 5.27	73.48 ± 5.39	70.94 ± 4.33	0.133
Height (m)	1.56 ± 0.08	1.53 ± 0.05	1.68 ± 0.07	**0.000**
Weight (kg)	68.55 ± 12.01	65.19 ± 9.63	83.43 ± 10.21	**0.000**
FM (%)	38.52 ± 6.43	39.79 ± 5.58	32.89 ± 7.11	**0.000**
BMI (kg/m^2^)	28.21 ± 3.84	27.93 ± 3.88	29.45 ± 3.55	0.219
MM (kg)	39.95 ± 8.20	37.16 ± 4.40	52.28 ± 9.80	**0.003**
**Physiological variables**				
SaO_2_ (%)	95.55 ± 1.53	95.40 ± 1.58^a^	96.17 ± 1.19	0.423
SBP (mmHg)	141.24 ± 17.02	139.16 ± 17.11^a^	150.25 ± 13.88	**0.041**
DBP (mmHg)	79.60 ± 8.81	78.56 ± 7.99^a^	84.13 ± 10.99	**0.048**
**Inspiratory strength and functional capacity**				
MIP (cmH_2_O)	52.89 ± 23.51	45.19 ± 16.38	86.92 ± 19.97	**0.000**
Distance walked at 6MWT (m)	557.26 ± 74.49	541.53 ± 66.97	626.71 ± 68.00	**0.000**
**Health-related quality of life**				
EQvas (%)	82.49 ± 17.58	82.26 ± 18.36	83.50 ± 14.30	0.894
EQindex (UA)	0.86 ± 0.13	0.86 ± 0.14	0.87 ± 0.09	0.704

a *Fifty-two women for these variables*.

*FM, fat mass; BMI, body mass index; MM, muscle mass; SaO_2_, arterial oxygen saturation; SBP, systolic blood pressure; DBP, diastolic blood pressure; MIP, maximum inspiratory pressure; 6MWT, 6-min walking test; EQvas, EuroQol-visual analog scales; EQindex, EuroQol index*.

### Correlations

Age was negative and moderately associated to HRQoL (although only to EQindex: *r* = −0.278; *p* = 0.024), MIP (*r* = −0.373; *p* = 0.002), and 6MWT (*r* = −0.438; *p* = 0.000). On the one hand, considering the whole sample (Table 2), while the MIP showed no other association, we found the expected moderate association between HRQoL and CRF (EQvas: *r* = 0.324, *p* = 0.009; EQindex: *r* = 0.312, *p* = 0.011). Introducing the covariate sex, this relationship was slightly modified: EQvas-6MWT decreased to small (*r* = 0.275; *p* = 0.028) and EQindex-6MWT increased a bit but remained moderated (*r* = 0.425; *p* =0.000). Adding sex and age as covariates, EQvas-6MWT association remained small (*r* = 0.319; *p* = 0.011) whereas EQindex-6MWT decreased to small (*r* = 0.264; *p* = 0.037).

**Table 2:** Association between the two components of the HRQoL questionnaire and the cardiorespiratory parameters MIP and 6MWT.

	**MIP**	**MIP^**s**^**	**MIP^**s+a**^**	**MIP^**w**^**	**MIP^**w(a)**^**	**MIP^**m**^**	**MIP^**m(a)**^**
EQvas	0.057	0.112	0.039	0.077	0.046	0.485	0.346
EQindex	−0.028	0.017	−0.107	−0.032	−0.118	**0.573**^**†**^	0.449
	**6MWT**	**6MWT**^**s**^	**6MWT**^**s+a**^	**6MWT**^**w**^	**6MWT**^**w(a)**^	**6MWT**^**m**^	**6MWT**^**m(a)**^
EQvas	0.324^**^	0.275^*^	0.319^*^	0.446^**^	0.443^***^	−0.241	−0.216
EQindex	0.312^*^	0.425^***^	0.264^*^	0.426^**^	0.333^*^	−0.133	−0.093

* p < 0.05;

** p < 0.01;

*** p < 0.005;

† *p = 0.051*.

*EQvas, EuroQol-visual analog scale; EQindex, EuroQol index; MIP, maximum inspiratory pressure; 6MWT, 6-min walking test; s, controlling by sex; s+a, controlling by sex + age; w, women group; w(a), women group controlling by age; m, men group; m(a), men group controlling by age*.

On the other hand, when we considered women and men separately, the association between 6MWT and both components of the HRQoL became moderated in women. However, these associations became non-significant in men. Furthermore, and noteworthy, in the men group, we observed a strong trend (*r* = 0.573; *p* = 0.051) toward a large and positive association between EQindex and MIP. Finally, considering age as covariate in each group, the relationship between HRQoL and 6MWT changed. Although the association remains moderate for both components of HRQoL, in this case, it is slightly higher for EQvas (*r* = 0.443; *p* = 0.001; EQindex: *r* = 0.333; *p* = 0.016).

**Figure 1:**
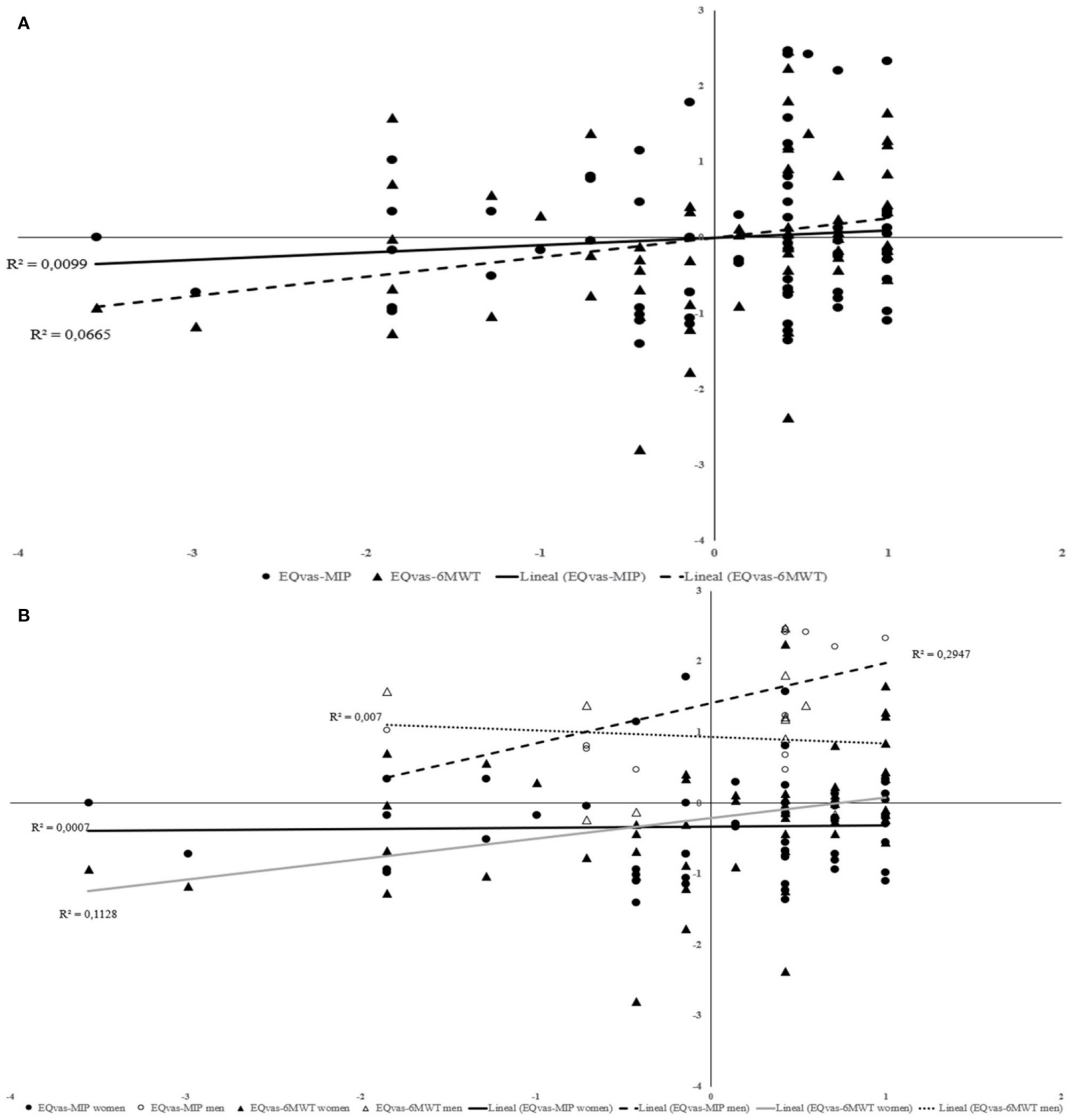
Relationship between EQvas and functional capacity; EQvas and inspiratory muscle strength for total sample **(A)** and considering women and men separately **(B)**.

*z*-Score scatterplot and *R*^2^ confirm these results. Figure 1A shows a small effect size for relationship between EQvas and 6MWT (*R*^2^ = 0.0665). Figure 1B shows a medium effect size for association EQvas-MIP in the men group (*R*^2^ = 0.2947). In the women group, there is a small effect size in EQvas-6MWT association (*R*^2^ = 0.1128).

**Figure 2:**
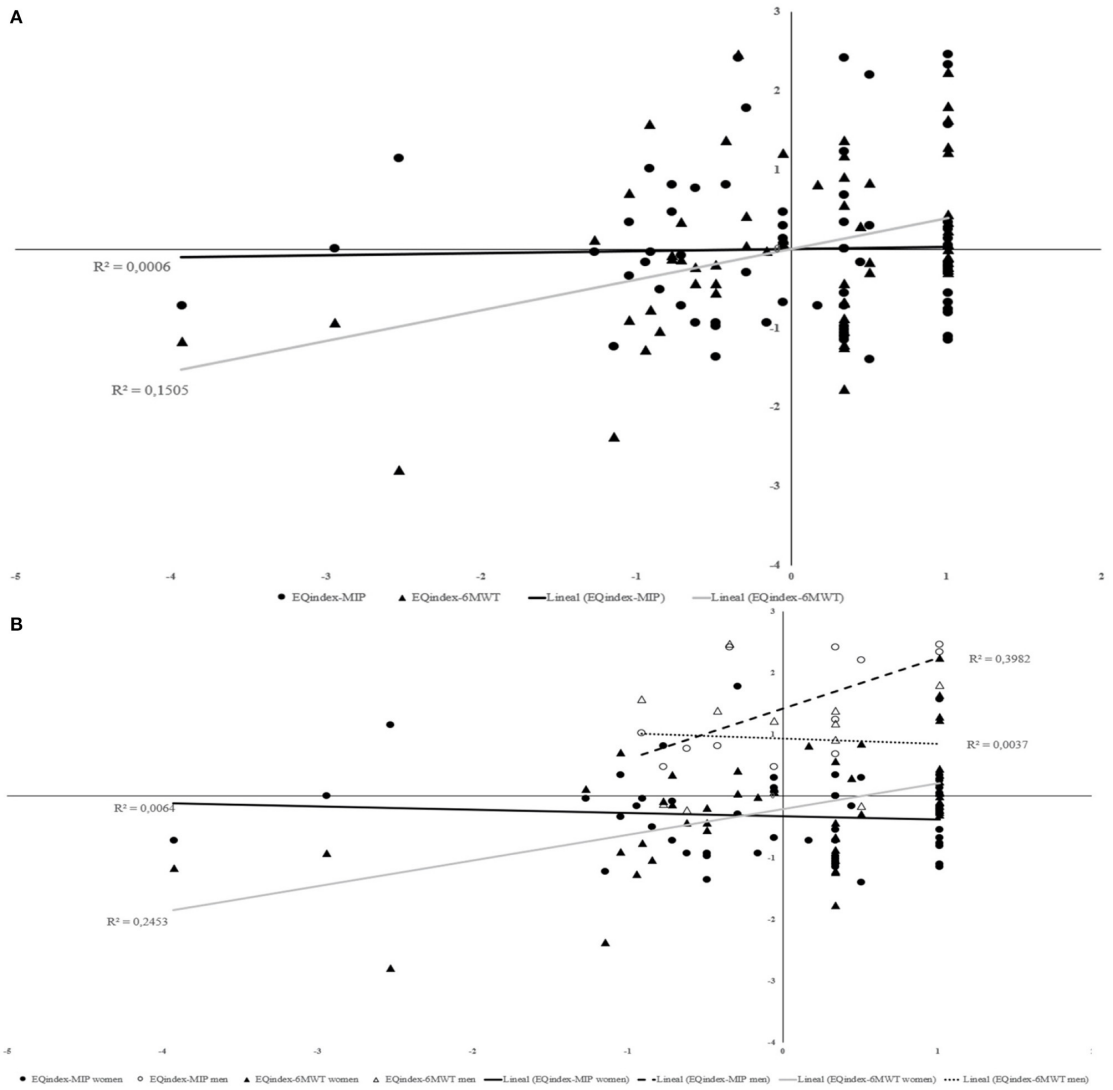
Relationship between EQindex and functional capacity; EQindex and inspiratory muscle strength for total sample **(A)** and considering women and men separately **(B)**.

Figure 2A shows a small effect size for relationship between EQindex and 6MWT (*R*^2^ = 0.1505). Figure 2B shows a medium effect size for association EQindex-MIP in men group (*R*^2^ = 0.3982). In the women group, there is a medium effect size in EQvas-6MWT association.

## Discussion

The purpose of this study was to analyze the association between HRQoL and CRF and inspiratory muscle strength in healthy active older adults, looking for similarities and differences. Although the relationship between HRQoL and CRF has been studied previously, up to our knowledge, no study has investigated the association between inspiratory muscle strength and HRQoL assessed by EQ-5D-5L questionnaire in this healthy population. Noteworthy, women presented inspiratory muscle strength values a 30.48% below the mean for their age, while men are 15.61% below the mean according to Black and Hyatt (1969). However, previous studies of the group pointed out that these lower MIP values were due to differences in the device of assessment (manual vs. electronic), leading to a false sense of low values (Roldán et al., 2019; Blasco-Lafarga et al., 2020). An idea supported by the results in Alvarenga et al. (2018).

Different limiting factors of the HRQoL have been previously studied in the older adults, although mainly in the context of pathological populations (Blanco-Reina et al., 2019; Ko et al., 2019). For example, with regard to cardiorespiratory responses, Wanderley et al. (2011) found a positive association between some-perceived health components of the Short Form-36 Health Survey (SF-36) and CRF in elderly with chronic pathologies, being this relationship independent of BMI, chronic condition, and education. More recently, Chung et al. (2017) found a positive and moderate association between HRQoL (measured through SF-36, another questionnaire to evaluate generic health-related quality of life) and physical function, but now in a sample of healthy seniors ranging from 65 to 84 years, giving some light to this relationship in the scope of healthy elderly.

Similarly, in a previous study of our research group, we found a positive and moderate association between EQindex and physical fitness in 58 healthy but sedentary older adults (Monteagudo et al., 2020). However, we did not find association between EQvas and physical fitness. In the current study, we found not only the expected moderate association between EQindex and CRF but also regarding EQvas. Besides, when considering age or sex + age, we obtained a reduction in the association between EQindex and CRF confirming that EQvas and EQindex behave differently in attending these covariates. On the one hand, this difference between studies might be attributed to the different assessments of physical fitness. In Monteagudo et al. (2020), physical fitness was obtained from the average of the standardized values of three well-known test: the Five-Times-Sit-to-Stand-Test, the 6-min walk, and the habitual gait speed in 6 m. In the current study, we only considered the 6-min walking test, increasing the importance of this outcome as a measure of the overall physical fitness. On the other hand, the persistent association between EQindex-CRF indicates that EQindex is a score influenced not only by CRF but also by strength and/or functional outcome. The fact that EQvas shows now an association not previously displayed, might be related to this increased importance of the CRF, pointing out that the worsening in this capacity affects the self-perception of mental or social health linked to EQvas. Moreover, EQindex might be more dependent on the physical deterioration and impairment process due to aging, compared with EQvas. Therefore, sex and also age are important in this population due to their heterogeneity, so the fact of not considering these variables in the analyses may hide some results and even lead to confusion in their practical applications.

On the other hand, it is known that active older adults have higher HRQoL values compared with inactive ones. Our data showed HRQoL values higher than expected in a healthy active population (Acree et al., 2006). In this sense, the Rejeski and Mihalko (2001) review concludes that physical activity can improve the perception of physical function and mental health, something that supports our results, because despite being a healthy sample, the fact of practicing physical activity on a regular basis makes them feel better. In this way, it could have been expected that elderly with greater inspiratory muscle strength would have better results in their HRQoL. However, this association is not fulfilled. Only the HRQoL component EQindex displays a strong trend to be associated with MIP in men, but the influence of age is even stronger and mediates it.

Renwick and Connolly (1996) investigated the association between respiratory function and quality of life, concluding that airway obstruction is a limiting factor; hence, people with preserved respiratory function have higher HRQoL values. In addition, it is very likely that the reduction in inspiratory muscle strength due to age may affect this association negatively. Notwithstanding, this deterioration it is not such in our healthy sample, and the reduction is not enough to cause dyspnea or fatigue, so the elderly does not perceive this loss of strength as a limitation. As a main finding, in absence of respiratory disease, the strength of the inspiratory muscles might be too local to be perceived as a limiting factor and affect the self-perceived health.

With regard to the CRF assessed through a walking test, walking has previously shown to integrate the valuation of physical, physiological, and cognitive function, with the participation of a large muscle mass, so our results are aligned with previous literature and confirm our hypothesis. Our results also confirm that sex differences and age are important mediators on the association of HRQoL and physical fitness, which was a second aim of the present study. The aging of both muscular systems is different, and although sarcopenia appears also in the respiratory muscles, it is a slower process compared with peripheral muscles (Shin et al., 2017) which are responsible for any physical activity and/or social relationships. Despite this, it is interesting to know that 6 weeks of inspiratory muscle training are able to improve HRQoL in relation to functionality domains (functional capacity and physical limitations) (Vilaça et al., 2019). Perhaps, this association depends not only on structural factors (a greater lower limb tone allows walking more, and therefore greater HRQoL) but also on nervous factors as a consequence of the metaboreflex (a greater inspiratory muscle tone results in walking more and a higher HRQoL).

This is the first study that has investigated the association between inspiratory muscle strength and HRQoL assessed by the EQ-5D-5L questionnaire in healthy older adults. Nonetheless, there are also some limitations that need to be outlined. First, the sample size should be bigger, at least in the men group, in order to generalize the results. Moreover, we performed this study in a sample of healthy older adults who performed the same multicomponent training program, so results are only representative of one type and dose of exercise. Whether the type of exercise and type of program can affect our findings and associations should be tested and deserves to be included in future studies. Finally, the design of the study should be highlighted like other limitations due to cross-sectional studies not showing evidence of a temporal relationship between outcomes.

To summarize, the fact that functional capacity is conditioned by sex (Botoseneanu et al., 2016) justifies that in our results, the association with HRQoL behaves differently in men and women. Although in most of the elderly physical exercise programs, women participation is higher than men, it would be a mistake to analyze the data without considering them separately since it can be misleading (Martínez del Castillo et al., 2009). Inspiratory muscle strength and CRF losses are greater in men compared with women and therefore the association of these variables with HRQoL behaves differently. Women confirm the hypothesized association between HRQoL, with slight differences either in the use of EQvas or EQindex, but not with the inspiratory strength. Conversely, men fail in this expected association, whatever the HRQoL index, but display a trend toward significance in the relationship between MIP and EQindex mediated by age. Noteworthy, the small sample size of the male group influencing these results needs further confirmation.

## Conclusion

In active healthy elderly, MIP do not seem to be a limiting factor for HRQoL when values of CRF are within the standard. MIP and HRQoL should be included in the assessment of exercise interventions because they provide information before significant losses in functional capacity occur. Besides, it is important to consider age and sex when analyzing these associations since older adults' population are characterized by being very heterogeneous. Keeping on research, these associations could help to improve training programs and their impact in this population.

## Data Availability Statement

The data analyzed in this study is subject to the following licenses/restrictions: The data is part of a thesis not yet published. Requests to access these datasets should be directed to Ainoa Roldán Aliaga ainoa.roldan@uv.es.

## Ethics Statement

The studies involving human participants were reviewed and approved by Research ethics committee of University of Valencia (H1506353751695). The patients/participants provided their written informed consent to participate in this study.

## Author Contributions

CB-L and AR contributed to conceiving, designing, performing the experiment, analyzing the data, drafting, and reviewing the article. PM contributed to performing the experiment, conceiving and interpreting the data, drafting, and revising the article. GS-S and AC performed most of the data analysis and drafting of the article. All authors contributed to the article and approved the submitted version.

## Conflict of Interest

The authors declare that the research was conducted in the absence of any commercial or financial relationships that could be construed as a potential conflict of interest.

## References

[B1] Acree L. S. Longfors J. Fjeldstad A. S. Fjeldstad C. Schank B. Nickel K. J.. (2006). Physical activity is related to quality of life in older adults. Health Qual. Life Outcomes 4:37. 10.1186/1477-7525-4-37 16813655 PMC1524938

[B2] Alvarenga G. M. D. Charkovski S. A. Santos L. K. D. Silva M. A. B. D. Tomaz G. O. Gamba H. R. (2018). The influence of inspiratory muscle training combined with the pilates method on lung function in elderly women: a randomized controlled trial. Clinics 73:e356. 10.6061/clinics/2018/e356 29924184 PMC5996441

[B3] Black L. F. Hyatt R. E. (1969). Maximal respiratory pressures: normal values and relationship to age and sex. Am. Rev. Respir. Dis. 99, 696–702. 10.1164/arrd.1969.99.5.696 5772056

[B4] Blanco-Reina E. Valdellós J. Ocaña-Riola R. García-Merino M. R. Aguilar-Cano L. Ariza-Zafra G.. (2019). Factors associated with health-related quality of life in community-dwelling older adults: a multinomial logistic analysis. J. Clin. Med. 8:1810. 10.3390/jcm8111810 31683766 PMC6912260

[B5] Blasco-Lafarga C. Cordellat A. Forte A. Roldán A. Monteagudo P. (2020). Short and long-term trainability in older adults: training and detraining following two years of multicomponent cognitive—physical exercise training. Int. J. Environ. Res. Public Health 17:5984. 10.3390/ijerph17165984 32824709 PMC7460235

[B6] Blasco-Lafarga C. Monteagudo P. Cordellat A. Roldán A. (2021). Inspiratory muscle strength, handgrip strength and muscle mass in active elderly women fuerza inspiratoria, fuerza de prensión y masa muscular en mujeres mayores activas. Rev. Int. Med. Cienc. Act. Fís. Deporte X. Available online at: http://cdeporte.rediris.es/revista/inpress/artfuerza1284e.pdf

[B7] Botoseneanu A. Allore H. G. Mendes de Leon C. F. Gahbauer E. A. Gill T. M. (2016). Sex differences in concomitant trajectories of self-reported disability and measured physical capacity in older adults. J. Gerontol. A Biol. Sci. Med. Sci. 71, 1056–1062. 10.1093/gerona/glw038 27071781 PMC4945890

[B8] Bouaziz W. Schmitt E. Vogel T. Lefebvre F. Leprêtre P. M. Kaltenbach G.. (2019). Effects of a short-term interval aerobic training programme with active recovery bouts (IATP-R) on cognitive and mental health, functional performance and quality of life: a randomised controlled trial in sedentary seniors. Int. J. Clin. Pract. 73:e13219. 10.1111/ijcp.13219 29963733

[B9] Bouaziz W. Schmitt E. Vogel T. Lefebvre F. Remetter R. Lonsdorfer E.. (2018). Effects of interval aerobic training program with recovery bouts on cardiorespiratory and endurance fitness in seniors. Scand. J. Med. Sci. Sports 28, 2284–2292. 10.1111/sms.13257 29969520

[B10] Chung P.-K. Zhao Y. Liu J.-D. Quach B. (2017). A canonical correlation analysis on the relationship between functional fitness and health-related quality of life in older adults. Arch. Gerontol. Geriatr. 68, 44–48. 10.1016/j.archger.2016.08.007 27620501

[B11] Ciprandi D. Bertozzi F. Zago M. Sforza C. Galvani C. (2018). Associations between objectively measured physical activity levels and physical fitness and health-related quality of life in elderly women. Sport Sci. Health 14, 183–191. 10.1007/s11332-018-0428-3

[B12] Crimmins E. M. Shim H. Zhang Y. S. Kim J. K. (2019). Differences between men and women in mortality and the health dimensions of the morbidity process. Clin. Chem. 65, 135–145. 10.1373/clinchem.2018.288332 30478135 PMC6345642

[B13] Cruz-Jentoft A. J. Bahat G. Bauer J. Boirie Y. Bruyère O. Cederholm T.. (2019). Sarcopenia: revised European consensus on definition and diagnosis. Age Ageing 48, 16–31. 10.1093/ageing/afy169 30312372 PMC6322506

[B14] de Oliveira L. D. S. S. C. B. Souza E. C. Rodrigues R. A. S. Fett C. A. Piva A. B. (2019). The effects of physical activity on anxiety, depression, and quality of life in elderly people living in the community. Trends Psychiatr Psychother 41, 36–42. 10.1590/2237-6089-2017-0129 30994779

[B15] DeFina L. F. Haskell W. L. Willis B. L. Barlow C. E. Finley C. E. Levine B. D.. (2015). Physical activity versus cardiorespiratory fitness: two (partly) distinct components of cardiovascular health? Prog. Cardiovasc. Dis. 57, 324–329. 10.1016/j.pcad.2014.09.008 25269066

[B16] Enright P. L. Kronmal R. A. Manolio T. A. Schenker M. B. Hyatt R. E. (1994). Respiratory muscle strength in the elderly. Correlates and reference values. Cardiovascular health study research group. Am J Respir Crit Care Med. 149, 430–438. 10.1164/ajrccm.149.2.8306041 8306041

[B17] Eriksen L. Grønbaek M. Helge J. Tolstrup J. (2016). Cardiorespiratory fitness in 16 025 adults aged 18–91 years and associations with physical activity and sitting time. Scand. J. Med. Sci. Sports 26, 1435–1443. 10.1111/sms.12608 26681406

[B18] Herdman M. Gudex C. Lloyd A. Janssen M. Kind P. Parkin D.. (2011). Development and preliminary testing of the new five-level version of EQ-5D (EQ-5D-5L). Qual. Life Res. 20, 1727–1736. 10.1007/s11136-011-9903-x 21479777 PMC3220807

[B19] Hopkins W. G. (2002). A scale of magnitudes for effect statistics. A new view of statistics. Psychology 3, 502, 411. Retrieved from: http://www.sportsci.org/resource/stats/effectmag.html

[B20] Huang C.-H. Yang G.-G. Wu Y.-T. Lee C.-W. (2011). Comparison of inspiratory muscle strength training effects between older subjects with and without chronic obstructive pulmonary disease. J. Formos. Med. Assoc. 110, 518–526. 10.1016/S0929-6646(11)60078-8 21783021

[B21] Ihász F. Schulteisz N. Finn K. J. Szabó K. Gangl J. Nagy D.. (2020). Associations between fitness levels and self-perceived health-related quality of life in community-dwelling for a group of older females. BMC Public Health 20, 1–9. 10.1007/s40520-018-1015-9 32799823 PMC7429675

[B22] Janssens J.-P. (2005). Aging of the respiratory system: impact on pulmonary function tests and adaptation to exertion. Clin. Chest Med. 26, 469–484. 10.1016/j.ccm.2005.05.004 16140139

[B23] Kim T. H. Jo M.-W. Lee S.-I. Kim S. H. Chung S. M. (2013). Psychometric properties of the EQ-5D-5L in the general population of South Korea. Qual. Life Res. 22, 2245–2253. 10.1007/s11136-012-0331-3 23224560

[B24] Ko H. Park Y.-H. Cho B. Lim K.-C. Chang S. J. Yi Y. M.. (2019). Gender differences in health status, quality of life, and community service needs of older adults living alone. Arch. Gerontol. Geriatr. 83, 239–245. 10.1016/j.archger.2019.05.009 31102926

[B25] Mandsager K. Harb S. Cremer P. Phelan D. Nissen S. E. Jaber W. (2018). Association of cardiorespiratory fitness with long-term mortality among adults undergoing exercise treadmill testing. JAMA Netw Open 1:e183605–e183605. 10.1001/jamanetworkopen.2018.3605 30646252 PMC6324439

[B26] Manini T. M. Clark B. C. (2012). Dynapenia and aging: an update. J. Gerontol. A Biol. Sci. Med. Sci. 67, 28–40. 10.1093/gerona/glr010 21444359 PMC3260480

[B27] Martínez del Castillo J. González Rivera M. D. Jiménez-Beatty Navarro J. E. Graupera Sanz J. L. Martín Rodríguez M. Campos Izquierdo A.. (2009). Los hábitos de actividad física de las mujeres mayores en España. Rev Int Cienc Deporte. 5, 81–93. 10.5232/ricyde2009.01407

[B28] Mateo D. C. Gordillo M. A. G. Olivares P. R. Adsuar J. C. (2015). Normative values of EQ-5D-5L for diabetes patients from Spain. Nutr. Hosp. 32, 1595–1602. 10.3305/nh.2015.32.4.9605 26545523

[B29] Mills D. E. Johnson M. A. Barnett Y. A. Smith W. H. Sharpe G. R. (2015). The effects of inspiratory muscle training in older adults. Med. Sci. Sports Exerc. 47, 691–697. 10.1249/MSS.0000000000000474 25116085

[B30] Monteagudo P. Cordellat A. Roldán A. Pesce C. Blasco-Lafarga C. (2020). Assessing health-related quality of life in older adults: EuroQol five-dimensional questionnaire vs the short form health survey. Sport Mont. 18, 117–120. 10.26773/smj.200609

[B31] Neder J. A. Andreoni S. Lerario M. C. Nery L. E. (1999). Reference values for lung function tests: II. Maximal respiratory pressures and voluntary ventilation. Braz. J. Med. Biol. Res. 32, 719–727. 10.1590/S0100-879X1999000600007 10412550

[B32] Orfila F. Ferrer M. Lamarca R. Tebe C. Domingo-Salvany A. Alonso J. (2006). Gender differences in health-related quality of life among the elderly: the role of objective functional capacity and chronic conditions. Soc. Sci. Med. 63, 2367–2380. 10.1016/j.socscimed.2006.06.017 16884840

[B33] Ran L. Jiang X. Li B. Kong H. Du M. Wang X.. (2017). Association among activities of daily living, instrumental activities of daily living and health-related quality of life in elderly Yi ethnic minority. BMC Geriatr. 17:74. 10.1186/s12877-017-0455-y 28330442 PMC5361829

[B34] Rejeski W. J. Mihalko S. L. (2001). Physical activity and quality of life in older adults. J. Gerontol. A Biol. Sci. Med. Sci. 56(suppl_2), 23–35. 10.1093/gerona/56.suppl_2.23 11730235

[B35] Renwick D. S. Connolly M. J. (1996). Impact of obstructive airways disease on quality of life in older adults. Thorax 51, 520–525. 10.1136/thx.51.5.520 8711681 PMC473599

[B36] Riddle E. S. Bender E. L. Thalacker-Mercer A. E. (2018). Expansion capacity of human muscle progenitor cells differs by age, sex, and metabolic fuel preference. Am. J. Physiol. Cell Physiol. 315, C643–C652. 10.1152/ajpcell.00135.2018 30110562

[B37] Rikli R. E. Jones C. J. (1999). Development and validation of a functional fitness test for community-residing older adults. J. Aging Phys. Act 7, 129–161. 10.1123/japa.7.2.129

[B38] Rikli R. E. Jones C. J. (2013). Development and validation of criterion referenced clinically relevant fitness standards for maintaining physical independence in later years. Gerontologist. 53, 255–267. 10.1093/geront/gns071 22613940

[B39] Roldán A. Cordellat A. Monteagudo P. García-Lucerga C. Blasco-Lafarga N. M. Gomez-Cabrera M. C.. (2019). Beneficial effects of inspiratory muscle training combined with multicomponent training in elderly active women. Res. Q. 90, 547–554. 10.1080/02701367.2019.1633009 31397649

[B40] Schoser B. Fong E. Geberhiwot T. Hughes D. Kissel J. T. Madathil S. C.. (2017). Maximum inspiratory pressure as a clinically meaningful trial endpoint for neuromuscular diseases: a comprehensive review of the literature. Orphanet J. Rare Dis. 12:52. 10.1186/s13023-017-0598-0 28302142 PMC5353799

[B41] Shaw S. Dennison E. Cooper C. (2017). Epidemiology of sarcopenia: determinants throughout the lifecourse. Calcif. Tissue Int. 101, 229–247. 10.1007/s00223-017-0277-0 28421264 PMC5544114

[B42] Shin H. I. Kim D.-K. Seo K. M. Kang S. H. Lee S. Y. Son S. (2017). Relation between respiratory muscle strength and skeletal muscle mass and hand grip strength in the healthy elderly. Ann. Rehabil. Med. 41:686. 10.5535/arm.2017.41.4.686 28971054 PMC5608677

[B43] Sperandio E. F. Guerra R. L. F. Romiti M. Gagliardi A. R. D. T. Arantes R. L. Dourado V. Z. (2019). Reference values for the 6-min walk test in healthy middle-aged and older adults: from the total distance traveled to physiological responses. Fisioter. Mov. 32:e003231. 10.1590/1980-5918.032.ao31

[B44] Strasser B. Burtscher M. (2018). Survival of the fittest: VO2max, a key predictor of longevity. Front. Biosci. 23, 1505–1516. 10.2741/4657 29293447

[B45] Sullivan G. M. Feinn R. (2012). Using effect size—or why the P value is not enough. J. Grad. Med. Educ. 4, 279–282. 10.4300/JGME-D-12-00156.1 23997866 PMC3444174

[B46] Tomás M. T. Galán-Mercant A. Carnero E. A. Fernandes B. (2018). Functional capacity and levels of physical activity in aging: a 3-year follow-up. Front. Med. 4:244. 10.3389/fmed.2017.00244 29376052 PMC5767296

[B47] Tsekoura M Kastrinis A Katsoulaki M Billis E Gliatis J. (2017) Sarcopenia and its impact on quality of life. in GeNeDis 2016. Advances in Experimental Medicine Biology Vol 987, ed Vlamos P. (Cham: Springer), 213–218. 10.1007/978-3-319-57379-3_19 28971460

[B48] Ventura-Clapier R. Moulin M. Piquereau J. Lemaire C. Mericskay M. Veksler V.. (2017). Mitochondria: a central target for sex differences in pathologies. Clin. Sci. 131, 803–822. 10.1042/CS20160485 28424375

[B49] Vilaça A. F. Pedrosa B. C. D. S. Amaral T. C. N. Andrade M. D. A. Castro C. M. M. B. D. França E. E. T.. (2019). The effect of inspiratory muscle training on the quality of life, immune response, inspiratory and lower limb muscle strength of older adults: a randomized controlled trial. Rev. Bras. Geriatr. Gerontol. 22:e190157. 10.1590/1981-22562019022.190157

[B50] Wanderley F. A. Silva G. Marques E. Oliveira J. Mota J. Carvalho J. (2011). Associations between objectively assessed physical activity levels and fitness and self-reported health-related quality of life in community-dwelling older adults. Qual. Life Res. 20, 1371–1378. 10.1007/s11136-011-9875-x 21380765

[B51] Wang D. X. Yao J. Zirek Y. Reijnierse E. M. Maier A. B. (2020). Muscle mass, strength, and physical performance predicting activities of daily living: a meta-analysis. J. Cachex. Sarcopen. Muscle Nerve 11, 3–25. 10.1002/jcsm.12502 31788969 PMC7015244

[B52] Wen Y. Wang D. Zhou M. Zhou Y. Guo Y. Chen W. (2019). Potential effects of lung function reduction on health-related quality of life. Int. J. Environ. Res. Public Health 16:260. 10.3390/ijerph16020260 30658477 PMC6352019

